# A facile hydrothermal approach for the density tunable growth of ZnO nanowires and their electrical characterizations

**DOI:** 10.1038/s41598-017-15447-w

**Published:** 2017-11-09

**Authors:** S. Boubenia, A. S. Dahiya, G. Poulin-Vittrant, F. Morini, K. Nadaud, D. Alquier

**Affiliations:** 1 Université François Rabelais de Tours, CNRS, GREMAN UMR 7347, 16 rue Pierre et Marie Curie, 37071 Tours Cedex2, France; 2 Université François Rabelais de Tours, INSA-CVL, CNRS, GREMAN UMR 7347, 3 rue de la Chocolaterie, CS 23410, 41034 Blois Cedex, France

## Abstract

Controlling properties of one-dimensional (1D) semiconducting nanostructures is essential for the advancement of electronic devices. In this work, we present a low-temperature hydrothermal growth process enabling density control of aligned high aspect ratio ZnO nanowires (NWs) on seedless Au surface. A two order of magnitude change in ZnO NW density is demonstrated *via* careful control of the ammonium hydroxide concentration (NH_4_OH) in the solution. Based on the experimental observations, we further, hypothesized the growth mechanism leading to the density controlled growth of ZnO NWs. Moreover, the effect of NH_4_OH on the electrical properties of ZnO NWs, such as doping and field-effect mobility, is thoroughly investigated by fabricating single nanowire field-effect transistors. The electrical study shows the increase of free charge density while decrease of mobility in ZnO NWs with the increase of NH_4_OH concentration in the growth solution. These findings show that NH_4_OH can be used for simultaneous tuning of the NW density and electrical properties of the ZnO NWs grown by hydrothermal approach. The present work will guide the engineers and researchers to produce low-temperature density controlled aligned 1D ZnO NWs over wide range of substrates, including plastics, with tunable electrical properties.

## Introduction

ZnO nanostructures such as nanowires (NWs), nanorods, and nanosheets have received considerable attention of the research community for the fabrication of electromechanical, electronic and optoelectronic devices, such as sensors^1^, energy harvesters^2,3^, field-effect transistors (FETs)^4–7^, and light emitting diodes^8,9^. The increased interest in ZnO nanomaterial has been largely driven by its excellent electrical and optoelectronic properties, including direct wide band-gap (3.37 eV)^10,11^, high exciton binding energy (60 meV)^11^, and high electron mobility (1 to 200 cm^2^/Vs) as well as high piezoelectric coefficient^1^(d_33_ ~12 pm/V). In the continual research effort for miniaturization of electronic devices, quasi one-dimensional (1D) ZnO nanowires (NWs), proved to be a potential candidate due to their unique properties, such as high electromechanical coupling factor and improved charge injection/extraction at metal-semiconductor junction^12^. Varieties of bottom-up approaches, including pulse laser ablation^13^, flamme transport approach^14–17^, vapor-liquid-solid^18^, electrochemical deposition^19^, hydrothermal and/or chemical bath deposition^2,6,11,20,21^ have been exploited for the synthesis of 1D ZnO NWs. However, most of the techniques are limited by their high temperature process that cannot be scaled up over large device area at very low cost. The need of an industrially scalable low-temperature ZnO NWs synthesis method has seen significant advancements towards the hydrothermal growth process. Hydrothermal growth (HTG) is a low temperature process where single-crystalline 1D material can be produced on various substrates, including plastics and textile fibers^22^. High-density ZnO NWs, perpendicular to the growth substrate, has often been reported employing HTG process^6^. However, perfect control of the ZnO NW’s density, aspect ratio and alignment with controlled electrical properties has rarely been reported. Moreover, it has been shown, both by simulations and experimental results, that the performances of electronic devices, such as piezoelectric nanogenerators, rely strongly on density and electrical properties of grown ZnO NWs^21,23^.

To control the density, uniformity and alignment of NWs, selective area HTG process has been used in the past. It has been demonstrated using different lithography technologies, including nanosphere lithography^24^, electron-beam (e-beam) lithography^25^, and laser interference lithography^26^. For instance, employing high resolution e-beam lithography, Consonni *et al*.^25^ reported position, vertical alignment, dimensions, and polarity controlled growth of ZnO NWs over large surface areas. However, adoption of these approaches are restricted due to many limitations such as: (i) use of expensive and complicated equipment added to cost and complexity of the final device, (ii) incompatibility with flexible substrates, and (iii) unscalability at industrial level. To mitigate these limitations, seedless density controlled growth of ZnO NWs, on Au surface, has been proposed eliminating the use of any lithography technique^27^. Such a growth approach has shown distinctive advantages, including: (i) low growth temperature enabling direct integration of NWs for the fabrication of functional devices onto organic substrates, (ii) tight control over size, orientation, and density of produced NW, and (iii) cost-effective. In most of the existing literature on seedless growth of ZnO NWs, the density is limited to one order variation. For instance, Xu *et al*. demonstrated one order change of NW density, by varying growth precursor concentration^27^. However, the exact growth mechanism leading to the variation of NW density is still unclear. Moreover, the consequence on the electrical properties, such as mobility and free charge density, related to the variation of growth nutrients and/or parameters to control the NW density, has not been performed.

A facile low temperature growth approach for 1D ZnO NWs with controlled morphology and density is a potential solution to master nanodevice fabrication cost. Furthermore, the direct integration of such quasi 1D nanostructures over metal electrodes not only reduces the fabrication cost but also the complexity of the fabrication process. A thin ZnO seed layer and/or ZnO nanoparticles is required for the growth of ZnO NWs^11^. Consequently, the presence of such ZnO layer between the metal electrode and NWs may increase the contact resistance, which has detrimental effect over the device performances due to poor charge injection / extraction across metal-semiconductor (MS) contact interface. The poor charge transport across MS contact can be improved by direct integration of ZnO NWs onto a metallic substrate such as Au metal electrode, like in the present study. Furthermore, we show a facile and effective HTG process to achieve high degree control over the ZnO NWs density on Au surface. More than two orders of NW density variation will be demonstrated by careful addition of NH_4_OH, as an additive, in the growth solution. Based on the experimental observations, we will hypothesize the mechanism leading to the density controlled growth of ZnO NWs. To follow the effect of NH_4_OH on the electrical properties of ZnO NWs, we will fabricate single ZnO nanowire field-effect transistors (NW-FETs) on Si/SiO_2_ substrates. The detailed statistical data of mobility and free charge density for ZnO NWs, grown with different NH_4_OH concentrations, will be presented.

## Experimental part

The ZnO NWs are grown by (HTG) process on (100) oriented Si wafers. A sample of 4 cm² rigid silicon is first cleaned in piranha solution (1:1 H_2_SO_4_ and H_2_O_2_) for 10 min followed by 2 min dip in hydrofluoric acid (50%) to remove the thin oxide formed during piranha cleaning and finally, rinsing in DI water. This clean step is followed by drying with nitrogen gas and a final baking step is performed at 200 °C to remove any adsorbed moisture before the metal deposition. A gold layer (~200 nm thick) is then deposited by direct current sputtering technique at room temperature. To improve the adhesion between gold and silicon, we deposit a layer of titanium (~100 nm) using the same technique. The reactant precursor consists of 1:1 ratio of zinc nitrate hexahydrate (Zn (NO_3_)_2_‚6H_2_O, 98%) and hexamethylenetetramine (HMTA). During the growth, the substrates are immersed facing down in a Teflon flask, sealed inside stainless steel autoclave reactor and placed in a preheated convection oven for 15 hours. The autoclave is taken out from oven and cools down naturally. The substrates are then thoroughly rinsed with flowing DI water and dried in N_2_ gas flow. In the experiments, the concentration of NH_4_OH is varied from 0 to 40 mM. The obtained ZnO NWs are examined under 10 kV of energy using Hitachi scanning electron microscope (SEM). The surface morphology of the as-deposited Au films is accessed using an atomic force microscopy (AFM). The AFM images are performed using a Nanoscope V (Bruker®) in tapping mode. The tip used is a non-conductive RTESP (MPP11100-10) probe with a resonance frequency of about 300 kHz and a spring constant of around 40 N/m.

ZnO NWs crystallinity is studied using x-ray diffraction (XRD) with CuKα_1_ radiation on the high resolution parallel beam diffractometer Bruker D8 discover. The scans are performed in the 2θ range from 25° to 50° at a scanning rate of 0.01° s^−1^. Room temperature Raman spectra of as grown ZnO NWs are obtained using a Renishaw Invia Reflex instrument. An excitation wavelength of 514.5 nm and a power of 1 mW is used. A lens of 100x magnification is used to focus the laser beam and, to collect the scattered light dispersed by a holographic grating with 2400 lines/mm. The diameter of the resulting laser spot is around 1 µm.

For the extraction of electrical properties of NWs, we have fabricated bottom-gate single ZnO NW-FET on highly doped Si substrates with 170 nm thick thermally grown SiO_2_. Fabrication of ZnO NW-FET devices is done using standard electron-beam lithography (EBL) process as follows: Following the growth of ZnO NWs by the HTG process, substrates are annealed in air ambient at 450 °C for 30 min. The annealed substrates are inserted into a vial filled with the desired solvent (isopropanol (IPA) in the present case). A brief agitation, on a sonic bath (5–10 sec), yields sufficient release and subsequent suspension of NWs in IPA solvent. The NWs solvent formulation is transferred directly (by drop-casting) onto the various device substrates using pipette. To define the source/drain (s/d) contacts on the single ZnO NW, EBL is performed. The s/d contact metallization is performed by electron beam evaporation at vacuum ≤10^−6^ mbar. The formation of ohmic s/d contacts for the devices is achieved using low work-function metals Ti/Al (100/400 nm). After metallization, the e-beam resist mask and unwanted metal layers are stripped from the surface of the substrates using acetone. This is followed by annealing the devices at 150 °C for 30 min on a hot plate in order to evaporate any residual solvent and/or water vapours from the NW surface and contact interface. This step improves the ohmicity of the metal-semiconductor (MS) contact interface, needed for the better extraction of semiconducting material properties. A Cascade Microtech Summit 11k probe stage equipped with a source measure unit (2636 A, a double channel source measuring unit by Keithley instrument) is used to perform current voltage measurements under dark ambient conditions. Tungsten metallic probes are used to contact NW-FET device electrodes for the electrical measurements.

## Results and Discussion

**Figure 1:**
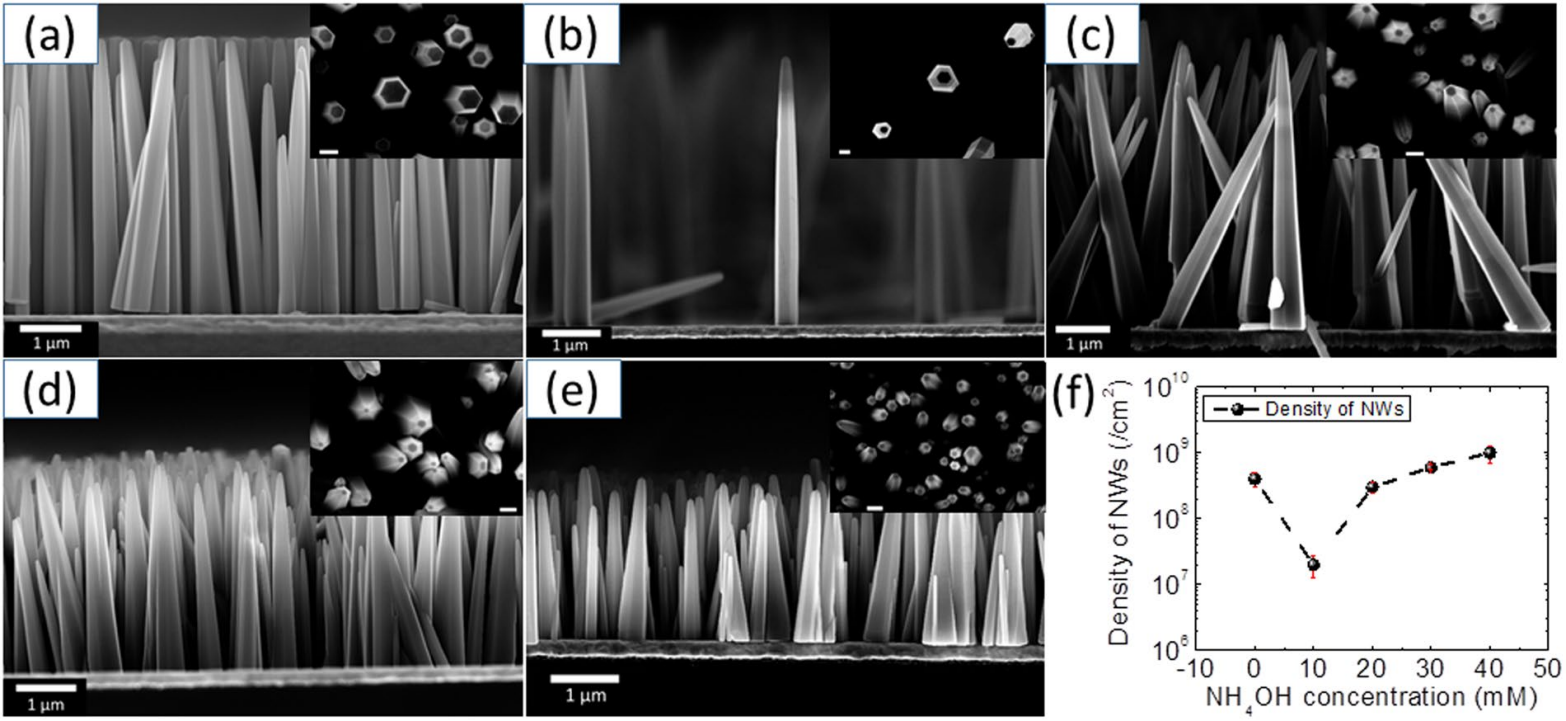
SEM images of NWs grown for different concentrations of ammonia: (**a**) 0 mM, (**b**) 10 mM, (**c**) 20 mM, (**d**) 30 mM, (**e**) 40 mM. The inset in each panel a-e shows the top view SEM image acquired from the same sample. The scale bar in the inset is 500 nm. (**f**) Panel shows the variation of density of NWs with the change in NH_4_OH concentration.

To control the density of ZnO NWs, ammonium hydroxide (NH_4_OH) has been used as an additive into the growth solution. The obtained growth results while varying NH_4_OH concentrations (from 0 to 40 mM by step of 10 mM) in the growth solution are presented in Fig. 1 (panel a-e); showing typical cross-sectional and top-view SEM images acquired from the ZnO NW samples. It can be seen from SEM images that the obtained NWs are almost perfectly aligned perpendicularly to the growth surface for all NH_4_OH concentrations. Using the SEM images, NW densities are extracted for all cases and the results are shown in Fig. 1 (panel f). From this graph, an increase in the NW density, defined as the number of NWs per square centimetre, can be detected when NH_4_OH concentration increases from 10 mM to 40 mM. As can be seen from Fig. 1f, an approximately two orders change in NW density can be obtained by careful control of NH_4_OH concentration in the growth solution. Going along with density, aspect ratio (AR) of the nanomaterial greatly determines / conditions their application in flexible electronics where high surface to volume ratio are needed for increased strain absorption. Hence, variation in NW’s AR, with the increase of NH_4_OH concentration, is also calculated using another set of SEM images. In order to have almost correct quantitative data for aspect ratio (due to their conical shape), we dispersed NW on another substrate and acquired SEM images for various NWs and the mean values are shown in Table 1. The diameter of ZnO NWs is determined from both tip (for AR_tip_) and base (for AR_base_) to take into account the possible change of their shape. It can be seen from Table 1, that there are very little variations of AR_base_, even though there is large variation in the length of NWs. On the other hand, a large variation of AR_tip_ is found with the change in NH_4_OH concentration, reaching a maximum value of 30 for 20 mM NH_4_OH. The obtained AR_tip_ values for all growth conditions are comparable to the other reported values for ZnO NWs grown on Au surface^28^. The growth mechanism leading to the large NW density variations with varying NH_4_OH concentrations is discussed in the following section.

**Table 1:** The aspect ratio of ZnO NWs with the variation of ammonium hydroxide.

Ammonia concentration mM	Average length µm	Aspect Ratio Tip	Aspect ratio Base
0	4.6	22 ± 6	9 ± 1
10	4.7	29 ± 4	10 ± 1
20	4.8	30 ± 4	12 ± 2
30	3.4	19 ± 3	10 ± 2
40	2.2	20 ± 3	10 ± 2

### Growth mechanism

**Figure 2:**
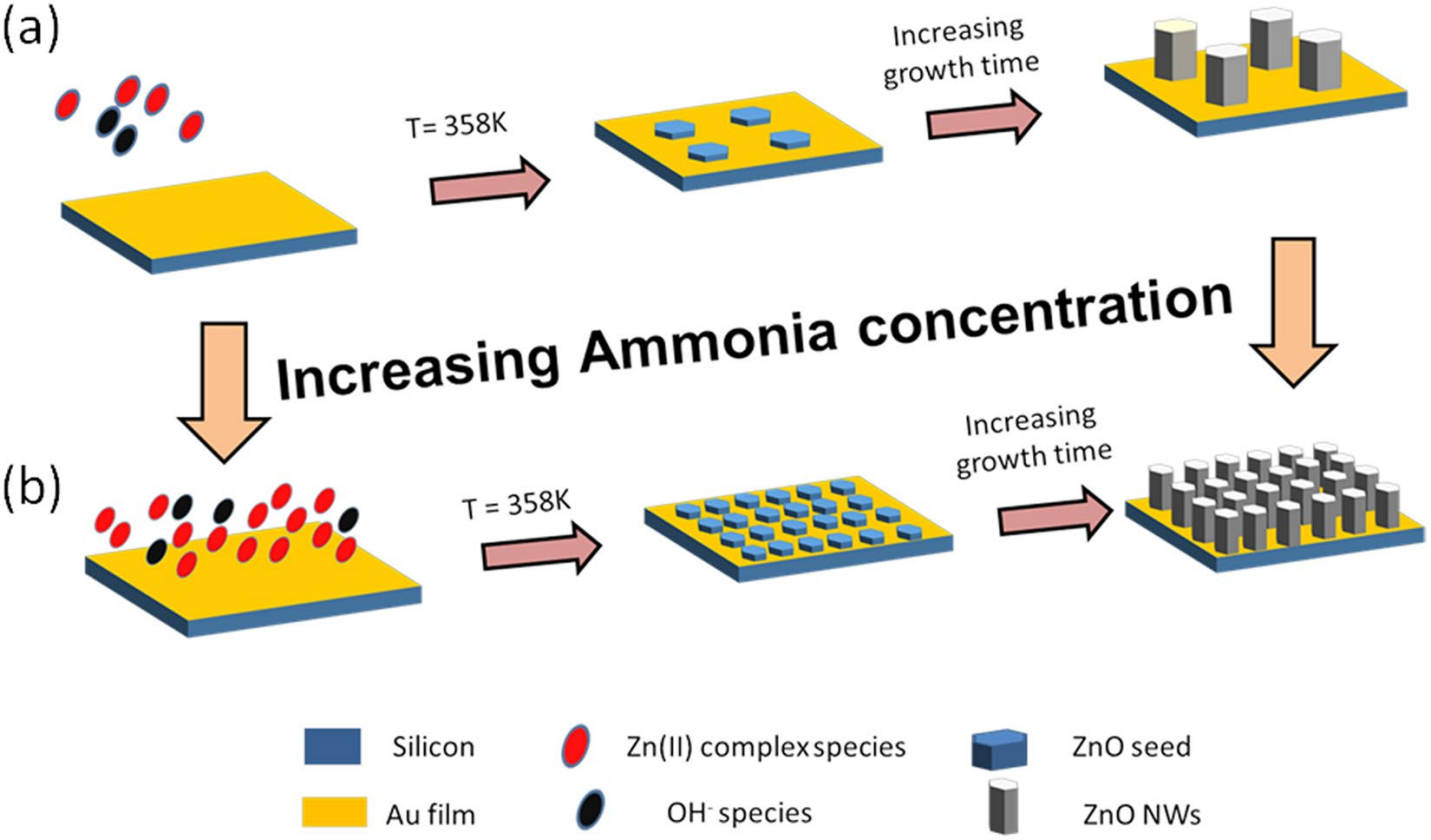
Growth mechanism for the density controlled growth of ZnO NWs: (**a**) low density, and (**b**) high density case.

Although the exact chemical reactions during the HTG of ZnO NWs is still unclear, we have hypothesized, based on the experimental observations, the growth mechanism for the density controlled growth of ZnO NWs on seedless growth substrates. The growth mechanism is schematically illustrated in Fig. 2, based on the analysis and growth models reported in the literature^20,29^. The following equations describe the dominant chemical reactions leading to the growth of ZnO NWs. First, HMTA and zinc nitrate salts can hydrolyze to produce ammonia and formaldehyde as well as zinc ions, respectively, as per the following equations:

1 $${({{\rm{CH}}}_{2})}_{6}{{\rm{N}}}_{4}+6{{\rm{H}}}_{2}{\rm{O}}\leftrightarrow 4{{\rm{NH}}}_{3}+6{{\rm{CH}}}_{2}{\rm{O}}$$

2 $${\rm{Zn}}{({{\rm{NO}}}_{3})}_{2}\to {{\rm{Zn}}}^{2+}+2{{{\rm{NO}}}_{3}}^{-}$$

Further, zinc nitrate ions can react directly with HMTA (Equation 3) and/or NH_3_ ions (Equation 4), produced in Equation 1, to provide positively charged Zn (II) complex:

3 $${({{\rm{CH}}}_{2})}_{6}{{\rm{N}}}_{4}+{{\rm{Zn}}}^{2+}\leftrightarrow {[{\rm{Zn}}({{\rm{C}}}_{6}{{\rm{H}}}_{12}{{\rm{N}}}_{4})]}^{2+}$$

4 $${{\rm{Zn}}}^{2+}+4{{\rm{NH}}}_{3}\leftrightarrow {\rm{Zn}}{({{\rm{NH}}}_{3})}_{4}^{2+}$$

On the other hand, negatively charged Zn (II) complexes can also be formed according to Equation 5:

5 $${{\rm{Zn}}}^{2+}+4{{\rm{OH}}}^{-}\leftrightarrow {\rm{Zn}}{({\rm{OH}})}_{4}^{2-}$$

Under given pH and temperature conditions, Zn (II) may exist primarily as Zn(OH)_4_^2−^ or Zn(NH_3_)_4_^2+^. ZnO NWs are formed on substrates, by the condensation (dehydratation) of these Zn (II) complexes (Equations 6–8):

6 $${\rm{Zn}}{({{\rm{NH}}}_{3})}_{4}^{2+}+{{\rm{H}}}_{2}{\rm{O}}\leftrightarrow {\rm{ZnO}}+4{{\rm{NH}}}_{3}+2{{\rm{H}}}^{+}$$

7 $${\rm{Zn}}{({\rm{OH}})}_{4}^{2-}\leftrightarrow {\rm{ZnO}}+{{\rm{H}}}_{2}{\rm{O}}+2{{\rm{OH}}}^{-}$$

8 $${[{\rm{Zn}}({{\rm{C}}}_{6}{{\rm{H}}}_{12}{{\rm{N}}}_{4})]}^{2+}+2{{\rm{OH}}}^{-}\leftrightarrow {\rm{ZnO}}+{{\rm{H}}}_{2}{\rm{O}}+{{\rm{C}}}_{6}{{\rm{H}}}_{12}{{\rm{N}}}_{4}$$

All these reactions occur in equilibrium and, hence, can be controlled by tuning the reaction parameters, such as precursor concentration, pH, and temperature.

The formation of ZnO nucleation growth sites, on Au surface, is a complicated task to accomplish. To initiate the nucleation process on Au surface, different techniques have been reported in the literature to introduce the negative charges over the Au surface to attract the positively charged zinc (II) complexes, formed during Equations 2 and 4. Some of these propose applying negative potential of 500 mV to the Au surface^29^ or activating Au surface using chemical treatments^30^. However, it is to note that, in the present case, we have employed no such extra step to initiate the nucleation process which eases the present growth approach.

**Figure 3:**
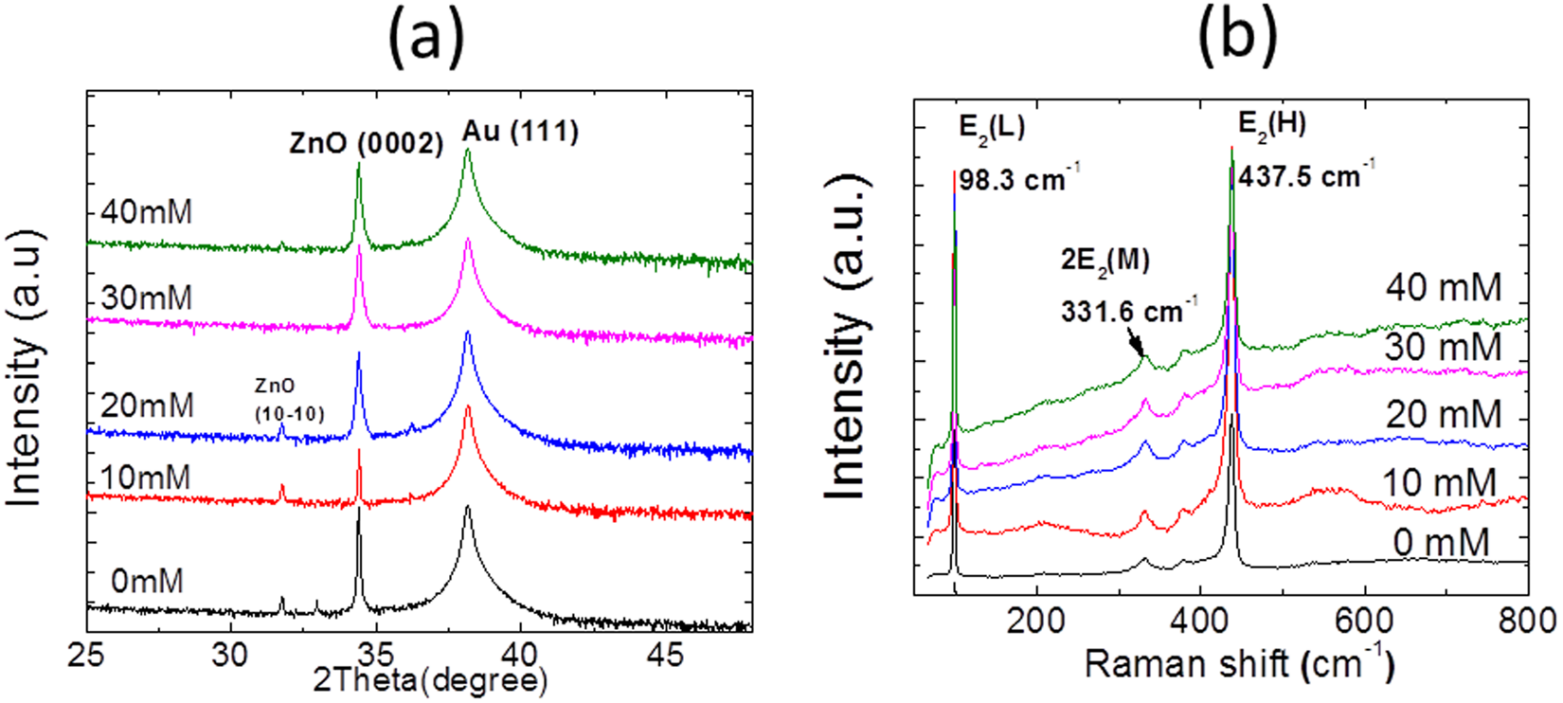
(**a**) XRD and (**b**) Raman spectroscopy acquired from ZnO NWs grown with different ammonium hydroxide concentrations.

In general, the surface roughness of the growth substrate have shown major effect on the orientation and quality of the NW produced^27^. In this work, the gold layer is (111) oriented as shown in Fig. 3a. The surface topography of the Au (111) sputtered film is characterized using atomic force microscopy (AFM) before starting the growth process. From the AFM characterization results (not shown here), a low RMS roughness value (~2 nm) is revealed, confirming the presence of a smooth Au (111) surface ideal for the growth of highly aligned ZnO NWs^27^. It is to note that no extra annealing step is carried to improve the crystalline quality of Au film. Most of the existing literature shows that the zinc oxide nanostructures are formed by the hydrolysis of zinc nitrate and HMTA^29^. Although the precise role of HMTA is still unclear, it is believed that it can act as a Lewis base with metal ions and a bidentate ligand capable of bridging two zinc(II) ions in solution^29^.

Moreover, according to the crystal growth theory, a requisite for the nucleation of a seed is the development and continuous presence of the supersaturation in the growth solution to provide a thermodynamic force to permit a spontaneous growth of the nuclei^20^. It has been reported that heating ammoniac solution containing dissolved zinc complexes provides this thermodynamic driving force for the ZnO synthesis^20^. In the present case, with very less NH_4_OH (10 mM), the pH of the growth solution is around 6.8. At this pH and temperature range (358 K), there are very few positively charges Zn (II) complex species present in the growth solution^20^. Such a low concentration of the ZnO precursors results into the low density of nucleation sites for the growth of ZnO NWs (Fig. 2a). When increasing the NH_4_OH concentration from 10 to 40 mM, the pH of the system increases from 6.8 to around 7. Although the change in pH is very low (between 6.8 and 7), there is an exponential change of the Zn (II) species in this pH regime^20^. In fact, this increase of pH leads to the increase of Zn (II) in the solution, which will largely decrease the Zn solubility and lead to a supersaturation large enough to initiate large number of nuclei both in the solution and onto the substrate. This causes a high concentration of the ZnO precursors to become localized at Au surface. Thus, enhanced nucleation sites could be achieved for the 40 mM NH_4_OH concentration (Fig. 2b).

The formation of the perfect hexagonal prism shaped NWs can be explained in accordance with the surface energies of the three lower index planes of ZnO, namely (0001),(000$\bar{1}$), and (1$\bar{1}$00). The Wurtzite ZnO has polar surfaces, i.e. (0001), (000$\bar{1}$) and non-polar surfaces (11$\bar{2}$0) and (10$\bar{1}$0). In general, under thermodynamic equilibrium conditions, the facet with higher surface energy is usually small in area, while the lower energy facets are larger^10^. The surface energy for the ZnO’s lower index planes are in the following order: (0001) > (11$\bar{2}$0) > (10$\bar{1}$0). Consequently, in the ZnO growth, the highest growth rate is along the c-axis and the large facets are usually (11$\bar{2}$0) and (10$\bar{1}$0). It has been proposed that the preferential adsorption of the negatively charged species onto the positively charged (0001) surface of ZnO leads to the anisotropic growth of rod like structures^20^.

A two orders of NW density control using NH_4_OH is impressive. However, addition of ammonia in the solution may have an adverse effect on the crystalline quality of ZnO NWs^31^. To follow the crystallinity of the obtained NWs, we have carried out XRD and Raman spectroscopy measurements, as shown in Fig. 3. The XRD spectra recorded from the various NW samples grown with different NH_4_OH concentrations are shown in Fig. 3a. As we can see, ZnO NWs show dominant peak at 2θ = 34.4°corresponding to (0002) plane reflections. This observation shows that ZnO NWs arrays exhibit strong preferential c-axis orientation vertically to the growth substrates. In addition, no significant shift is observed in the dominant ZnO related peak (0002) with increasing the ammonia concentration, indicating the absence of any additional induced stress in the NWs. These observations lead us to conclude that there is no distortion of the ZnO lattice parameter with the NW density change. Moreover, there is no other extra peak related to any impurities which indicates that the NWs, for different NH_4_OH concentrations, have pure wurtzite crystal structure. The additional dominant peak appearing at 2θ = 38.3° is assigned to Au (111). The crystalline quality of as-grown ZnO NWs is further confirmed by micro-Raman measurements, as shown in Fig. 3b. The Micro-Raman spectroscopy is a powerful characterization tool to detect level of point defects, doping and change of crystalline structure of the obtained material. As observed in the Fig. 3b, all the spectra recorded for various NH_4_OH concentrations at room temperature, exhibit the Raman modes typical of the wurtzite crystal structure, as indicated by the XRD measurements. The two dominant peaks centred at 98.3 and 437.5 cm^−1^ are successfully assigned to the two non-polar first-order Raman active E2 (low) and E2 (high) modes, described by the Raman selection rules for wurtzite ZnO (with C6v point group symmetry). Similarly to XRD measurements, the ZnO frequency Raman modes remain unchanged whatever the NH_4_OH concentration of this study and no additional mode is detected. This confirms the formation of the wurtzite structure. Moreover, both the peaks are found to show very low full width half maximum (FWHM) values of 3 and 7, respectively. Such low FWHM values for NWs grown with all NH_4_OH cases, suggest the production of high ZnO crystal quality^32^. The peak appearing at 332 cm^−1^ is the second order scattering from phase boundaries of ZnO.

**Figure 4:**
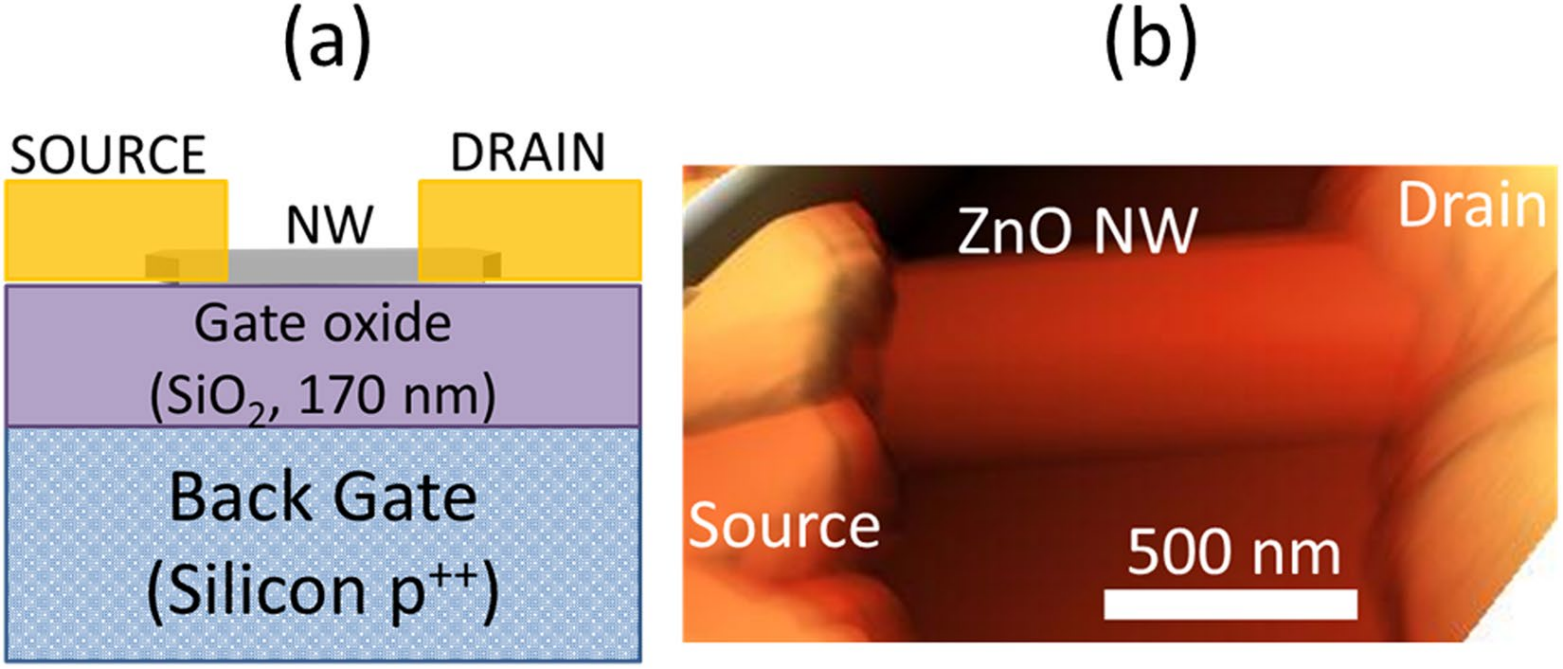
(**a**) Schematic and (**b**) atomic force microscopy image of a typical NW-FET fabricated on 170 nm thick SiO_2_.

It is indeed important to control the electrical properties of the 1D nanomaterial for the advancement of electronic, optoelectronic and electromechanical devices, such as piezoelectric nanogenerators^2^. It is well known that ZnO material, irrespective bulk or nanostructures, are always unintentionally n-type doped. The cause of this unintentional n-type conductivity has been widely discussed in the literature, and has often been attributed to the presence of native point defects, such as oxygen vacancies and zinc interstitials^33,34^. The addition of ammonia during the hydrothermal growth process has shown to introduce additional point defects observed using optical characterization techniques, such as photoluminescence which will affect the electrical properties of the material^31^. Nevertheless, no such statistical study has been performed to evaluate the effect of NH_4_OH on the electrical properties of the nanomaterial. Hall-effect measurements is the commonly used technique to study the charge transport in thin-films or bulk, however in case of 1D NWs, it is very complicated to implement Hall measurements due to the limitation of the size of the material. In general, for 1D nanostructures, electrical characterization of the material is performed through the fabrication of single NW field-effect transistors (NW-FETs)^6^. In the present work, to evaluate the effect of NH_4_OH on the electrical parameters of the NWs, bottom-gate single NW-FETs are fabricated on Si/SiO_2_ substrates. Figure 4 shows the schematic and typical atomic force microscopy (AFM) image of the fabricated NW-FET device. It can be seen, using the AFM image, that the NW is perfectly covered by source/drain (s/d) electrodes, a feature needed for the efficient charge transport across metal-semiconductor interface.

**Figure 5:**
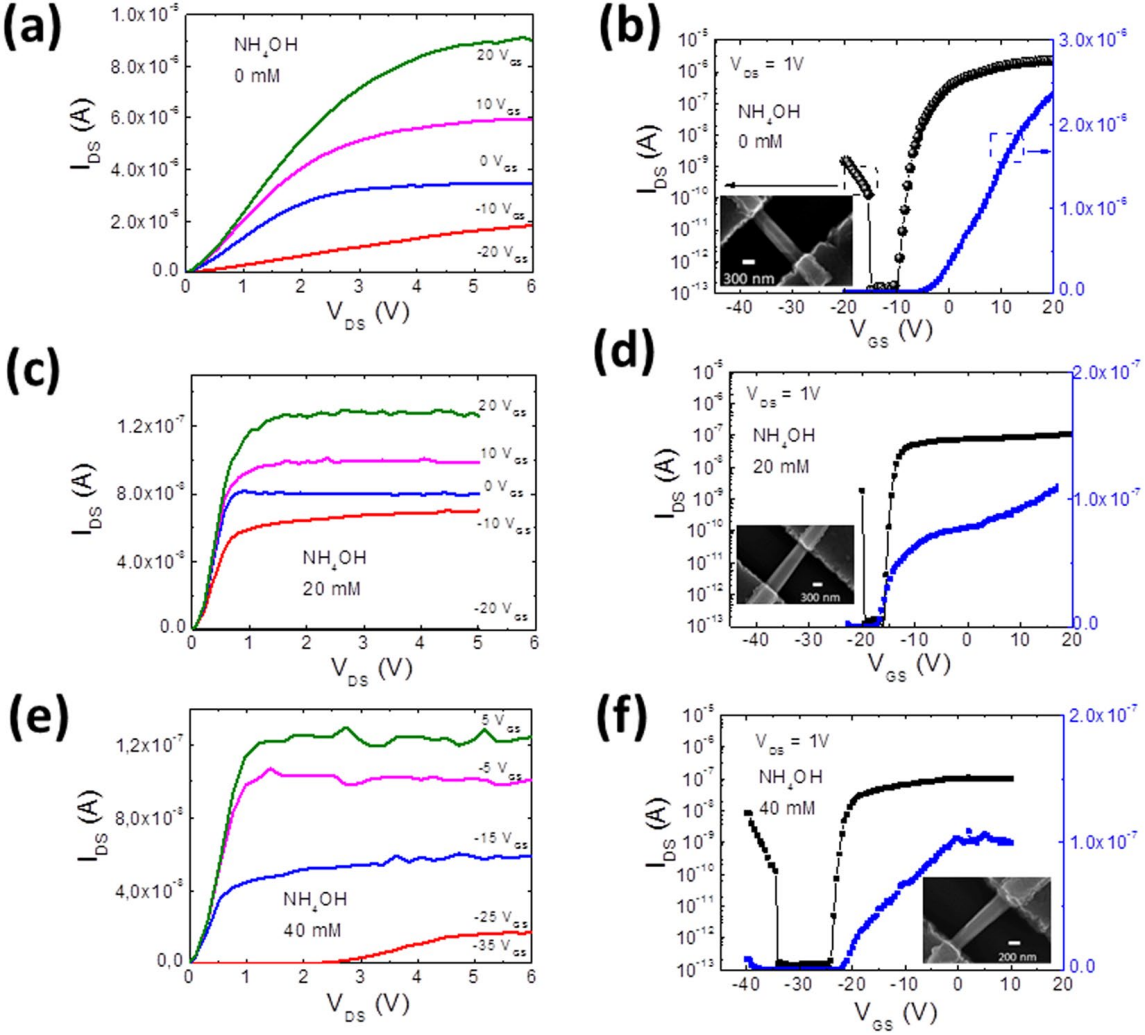
Electrical characterization results for ZnO NW-FETs fabricated using NWs grown using different NH4OH concentrations: (**a**,**b**) 0 mM, (**c**,**d**) 20 mM and (**e**,**f**) 40 mM. The inset shows the SEM image of the NW-FET.

To evaluate the effect of NH_4_OH on the electrical properties, we fabricate single NW-FETs using NWs grown with 0, 20 and 40 mM of ammonium hydroxide. It is to note that all the NWs are thermally annealed under same conditions (450 °C in air for 30 min) in order to have field-effect modulation of current, that will enable the extraction of electrical parameters^6^. The resulting electrical characterization results, output and transfer curves, for different NH_4_OH concentrations (0, 20, and 40 mM), are shown in Fig. 5 along with their respective device SEM images. The output characteristics are shown in Fig. 5a,c and e for 0, 20 and 40 mM concentration of NH_4_OH, respectively. The observed increase in drain-source current (*I*_DS_) with the incremental increase of gate-source voltage (*V*_GS_) towards the positive values, in the output scans, shows the n-channel behaviour of the NW-FET devices for all cases. Note that the output scans also demonstrate a linear dependence of *I*_DS_ with increasing drain-source voltage (*V*_DS_) (at *V*_DS_ *≤ *0.1 V), and nicely saturating at higher *V*_DS_ values confirming a tight gate control over the NW channel, a feature needed for the extraction of the near real electrical parameter values of the semiconductor material. To obtain the transfer scans, the *V*_GS_ is swept from negative towards positive *V*_GS_ at a fixed *V*_DS_ of 1 V. The resulting curves are shown in Fig. 5 panel’s b (0 mM), d (20 mM) and f (40 mM). From these transfer scans, it can be seen that increasing V_GS_ towards positive values resulted to an increase in *I*_*DS*_. This device behaviour suggests an n-channel accumulation-type FET. A linear extrapolation of *I*_DS_ to *V*_GS_ interception reveal the threshold voltage (*V*_TH_) of the device. Using the transfer characteristics, important electrical data of the semiconductor NWs, such as mobility and charge density can be extracted using the following equations:^6^

9 $$\mathrm{Field}-\mathrm{effect}\,{\rm{mobility}},\,{\mu }_{FE}=\frac{{g}_{m}\times {L}^{2}}{{V}_{DS}\times {C}_{NW}}$$

10 $${\rm{Free}}\,{\rm{charge}}\,{\rm{density}},\,{n}_{e}=\frac{{C}_{NW}{V}_{TH}}{2\pi {r}^{2}qL}$$

Where g_m_ is the transconductance (d*I*_DS_/ d*V*_GS_), *L* is the channel length, *r* is the radius of NW, q is the elementary charge and C_NW_ is the gate capacitance given by:

11 $${C}_{NW}=\frac{2\pi {\varepsilon }_{0}{\varepsilon }_{ox}L}{{\cosh }^{-1}(\frac{{r}_{NW}+{t}_{ox}}{{r}_{NW}})}$$

where ε_0_ is the absolute permittivity, ε_ox_ is the silicon oxide permittivity (3.9) and t_ox_ is SiO_2_ thickness (170 nm).

To have the statistical data distributions for charge density and mobility values, we have fabricated 5 NW-FET devices for each NH_4_OH concentration. The data distribution is shown in Table 2. It can be seen from Table 2, that all the NW-FET devices showed impressive on/off current ratio which may find them applications in digital electronics as switches. The precise values of mobility and charge density are difficult to extract using FET device configuration which generally shows large data variations from device to device^4^. The large variations of electrical parameters, such as mobility, may be due to the quality of the s/d contact interfaces, which can contain some insulating surface layers. Such layers can arise from: (1) the presence of residual resists from the assembly stage and/or (2) unfavorable chemical reactions between metal s/d contacts and ZnO surface^35^. As can be seen from Table 2, there is large V_TH_ variation from device to device in the present case also which affects the extraction of precise value for free charge density (Equation 10). We acknowledge that, in the present work, we have not mastered the threshold voltage of ZnO NW-FETs, where the value varies largely. In thin film transistors and/or nanomaterial based FETs, which work in depletion/accumulation mode, V_TH_ is influenced by many factors, such as charge density in the nanomaterial, energy bands at both s/d metal-semiconductor contact interface, dielectric/semiconductor interface quality and adsorbed species on semiconductor channel^32^. From Table 2, the average V_TH_ values of NW-FET devices fabricated for each NH_4_OH concentration become more negative when we introduce NH_4_OH in the growth solution. The additions of NH_4_OH is supposed to introduce extra point defects in the NWs which degrade the high quality of the nanomaterial produced and hence increase the charge density in the NWs. It is also to note from Table 2 that FET devices fabricated with NWs obtained by addition of NH_4_OH showed large V_TH_ variation ( ± 18 V) compared to the one without ammonium hydroxide ( ± 4.7). Such a large variation in V_TH_ can arise from the creation of extra defect carrier trap centres at the metal-semiconductor (MS) contact, which will degrade the quality of the charge transport in the material. Investigating the exact cause of such device to device V_TH_ variation is beyond the scope of the present manuscript, therefore this V_TH_ variation will be more deeply investigated in a future work by post-treatments on the FET devices such as annealing in oxygen ambient and/or ozone treatment^32,36^. However as all the fabrication steps for different devices are performed under identical conditions, therefore it is fair to compare the NW electrical parameters values such as mobility and free charge density, obtained from different devices.

**Table 2:** Key parameters extracted from NW-FET fabricated with NWs grown with varying ammonium hydroxide concentration.

NH_4_OH concentration (mM)	V_TH_ variation (V)	On/Off current ratio	Field-effect mobility, µ_FE_ (cm^2^/Vs)	Free charge density, n_e_ (/cm^3^)
0	−5.4 ± 4.7	10^6^–10^7^	3.8 ± 3.3	4.3 ± 3.9 × 10^16^
20	−20.7 ± 18	10^5^–10^6^	2 ± 2	8.3 ± 4 × 10^16^
40	−13 ± 18	10^5^–10^7^	0.35 ± 0.3	2 ± 1 × 10^17^

**Figure 6:**
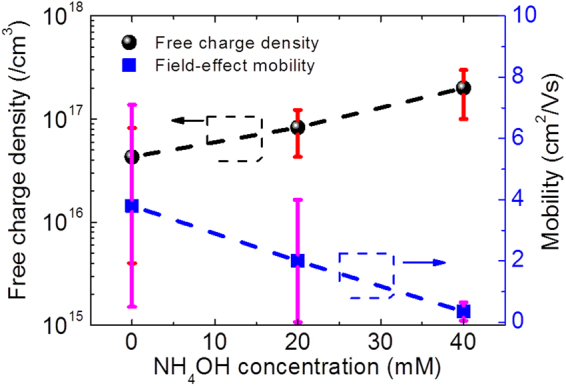
Graph depicting the variation of mobility and free chare density with the addition of NH_4_OH in the growth solution.

The extracted average mobility and charge density values with possible errors are shown in Fig. 6. As it can be seen from this set of data, with increasing NH_4_OH concentration, the free charge carrier density increases from 4.3 × 10^16^ to 2 × 10^17^ cm^−3^ while field-effect mobility decreases from 3.8 to 0.35 cm^2^/Vs as the NH_4_OH concentration increases from 0 to 40 mM. These observations suggest that with increasing amount of NH_4_OH concentration in the growth solution, the amount of free charge density increases inside ZnO NWs. The increase of free charge density can be correlated with the increase of point defects, such as oxygen vacancies or Zn interstitials. These defects, commonly observed on ZnO nanowires grown by hydrothermal method, are enhanced with the addition of NH_4_OH, as it is reported in literature^31^. Free charge density values, deduced from the electrical characterization performed on the ZnO nanowires, confirm this trend with increasing NH_4_OH concentration, as shown in Fig. 6.

## Conclusion

In summary, we have developed a low-cost and scalable bottom-up growth process of ZnO NWs, with the control of both NW density and their electrical properties. From the experimental observations, we found that the concentration of ammonium hydroxide plays a crucial role in controlling the NW area density. With a careful manipulation of the amount of ammonium hydroxide in the solution, we demonstrated that the NW density can be controlled over two orders of magnitude. Based on the obtained results, we proposed the growth mechanism governing the density controlled synthesis of ZnO NWs. It is hypothesized that the amount of ammonium hydroxide has a direct effect over the concentration of Zn (II) complexes which will largely affect the Zn solubility in the solution. Consequently, the supersaturation of the growth solution can be controlled and so, the number of nuclei over the substrate. Furthermore, the effect of NH_4_OH addition over the electrical properties of the ZnO NWs was evaluated by fabricating single NW-FETs. It was observed that the free charge carrier density increases from 4.3 × 10^16^ to 2 × 10^17^ cm^−3^ while field-effect mobility decreases from 3.8 to 0.35 cm^2^/Vs, as the NH_4_OH concentration increases from 0 to 40 mM, hinting the creation of extra point defects with the addition of NH_4_OH in the growth solution. This study presents a useful method to control the morphology, density and electrical properties of ZnO NWs obtained by hydrothermal growth on seedless Au substrates. Such a work will pave the way for ZnO NW’s efficient use into the development of flexible electronic and electromechanical devices, such as piezoelectric nanogenerators.
